# Deep Learning Pipeline for Automated Cell Profiling from Cyclic Imaging

**DOI:** 10.21203/rs.3.rs-3745061/v1

**Published:** 2023-12-18

**Authors:** Christian Landeros, Juhyun Oh, Ralph Weissleder, Hakho Lee

**Affiliations:** 1. Center for Systems Biology, Massachusetts General Hospital, Boston, MA 02114, USA; 2. Harvard-MIT Program in Health Sciences and Technology, Massachusetts Institute of Technology, Cambridge, MA 02139, USA; 3. Department of Radiology, Massachusetts General Hospital, Harvard Medical School, Boston, MA 02114, USA; 4. Department of Systems Biology, Harvard Medical School, Boston, MA 02115, USA

## Abstract

Recent advances in microscopy allow scientists to generate vast amounts of biological data from a single biopsy sample. Cyclic fluorescence microscopy, in particular, enables multiple targets to be detected simultaneously. This, in turn, has deepened our understanding of tissue composition, cell-to-cell interactions, and cell signaling. Unfortunately, analysis of these datasets can be time-prohibitive due to the sheer volume of data. In this paper, we present CycloNET, a computational pipeline tailored for analyzing raw fluorescent images obtained through cyclic immunofluorescence. The automated pipeline pre-processes raw image files, quickly corrects for translation errors between imaging cycles, and leverages a pre-trained neural network to segment individual cells and generate single-cell molecular profiles. We applied CycloNET to a dataset of 22 human samples from head and neck squamous cell carcinoma patients and trained a neural network to segment immune cells. CycloNET efficiently processed a large-scale dataset (17 fields of view per cycle and 13 staining cycles per specimen) in 10 minutes, delivering insights at the single-cell resolution and facilitating the identification of rare immune cell clusters. We expect that this rapid pipeline will serve as a powerful tool to understand complex biological systems at the cellular level, with the potential to facilitate breakthroughs in areas such as developmental biology, disease pathology, and personalized medicine.

## INTRODUCTION

With improved microscopy instrumentation, researchers increasingly produce a wealth of biological data from imaging experiments. Either by mechanical scanning or computational post-processing (e.g., Fourier ptychography, diffraction microscopy), millimeter-scale fields of view can be digitized in sub-cellular detail^1–3^. Moreover, the continued development of fluorescent and colorimetric probes allows numerous sub-cellular structures (e.g., proteins, nucleic acids) to be quantified and spatially visualized. This multi-parametric capacity has been critical to unveiling heterogeneity in biological samples and identifying cell subpopulations with key biological profiles^4–10^. One enabling technology is cyclic multiplexed immunofluorescence microscopy (mIF); through repeated staining, destaining (or quenching), and re-staining of biological samples, this approach significantly expands the number of targets that can be simultaneously detected in the same sample. A considerable number of different technologies of mIF have been developed based on chemical or optical quenching^11–17^. Resulting molecular data have deepened our understanding of tissue composition, cell-to-cell interactions, and cell signaling^18, 19^.

Analyzing large cyclic-imaging datasets, however, presents computational challenges. For example, imaging a single tissue sample already generates vast amounts of visual data, including i) hundreds of fields of view (FOVs), ii) multiple staining rounds per field of view (often 20–40), and iii) corresponding background intensity images for each marker. As a result, standard steps such as image registration between cycles require intensive computation with standard imaging tools. Similarly, manual cell segmentation on this scale would require several person-hours per sample, severely limiting clinical applications. Off-the-shelf cell segmentation tools (e.g., CellProfiler) reduced some of the workloads, but noise introduced through stain variation and sample debris made consistent performance across samples difficult.

Here, we present a computational framework to analyze multi-channel, large FOV cellular images. Termed CycloNET, this framework i) consolidates, aligns, and processes several gigapixels of fluorescent microscope images, ii) segments individual cells and generates their multi-dimensional molecular profiles, and iii) produces visual representations that succinctly summarize cell profiles. As a representative example, we applied CycloNET to profile immune cells in fine-needle aspirate (FNA) samples of head and neck squamous cell carcinoma (HNSCC) patients. CycloNET significantly reduced analysis time when compared to standard computational imaging tools and manual cell segmentation. Further, our pipeline facilitated the identification of rare immune cell clusters, which could be instrumental to future research in cancer management. The developed framework would be readily adapted for applications involving other cell types or tumor structures (i.e., tumor cells, lymphatic or vascular structures). Combined with cyclic imaging, we expect CycloNET will facilitate the development of improved diagnostic and prognostic metrics from complex information-rich imaging data.

## RESULTS AND DISCUSSIONS

### CycloNET pipeline

**Figure 1.**
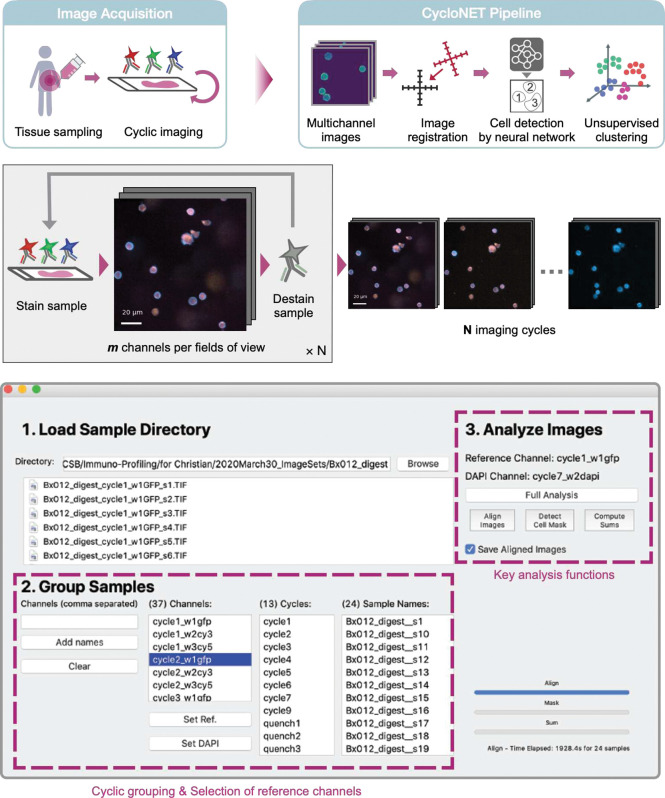
Overview of CycloNET analysis pipeline. **(A)** Schematic for computationally assisted biopsy analysis. After immunofluorescent images are collected via cyclic staining, CycloNET automatically aligns images from different cycles, produces a single-cell segmentation mask, and generates 2-dimensional representations for multiplexed biomarker data. **(B)** Cyclic staining protocol. A sample is stained, and several fields of view, *m*, are captured by a microscope. The fluorescent signal is then quenched, and the background “quenched signal” is imaged. This process is repeated *N* times to produce the full dataset. **(C)** Analysis user interface. A simple user interface was developed to group sample images and execute the CycloNET modules described above.

Figure 1A shows CycloNET’s overall workflow. The input data are raw fluorescent images obtained through cyclic immunofluorescent microscopy. The computational pipeline collates acquired multichannel images, performs image registration, detects individual cells, and presents summary statistics. In the current work, we used images generated by the fast analytical screening technique (FAST) as previously reported^18, 19^; FAST uses custom-designed antibody probes to cyclicly image 20–40 molecular markers within an hour^18, 19^ (Fig. 1B). A given specimen underwent rounds of imaging and quenching cycles; at each cycle, several FOVs were captured, resulting in up to 5 gigapixels of visual information per sample. We implemented a graphical user interface to streamline image analysis (Fig. 1C). Under the hood, it was equipped with i) a preprocessor that automatically grouped images by cycle and FOV, ii) a registration module that detected translations between imaging cycles, and iii) a pre-trained neural network for task-specific cell segmentation.

### Image registration

Between fluorescent staining and destaining cycles, sample slides were manually removed and remounted onto the microscope, introducing spatial offsets. Though negligible for tissue-level assessments, these shifts required careful correction for accurate single-cell metrics. Standard imaging software for spatial alignment faced challenges, including (i) algorithms that were suboptimal for large image sets and (ii) errors in registration due to sample debris, low cellularity, or low signal-to-noise ratio (SNR). We addressed these drawbacks in our custom-designed processing pipeline, optimized for cyclic imaging.

### Pre-processing

**Figure 2.**
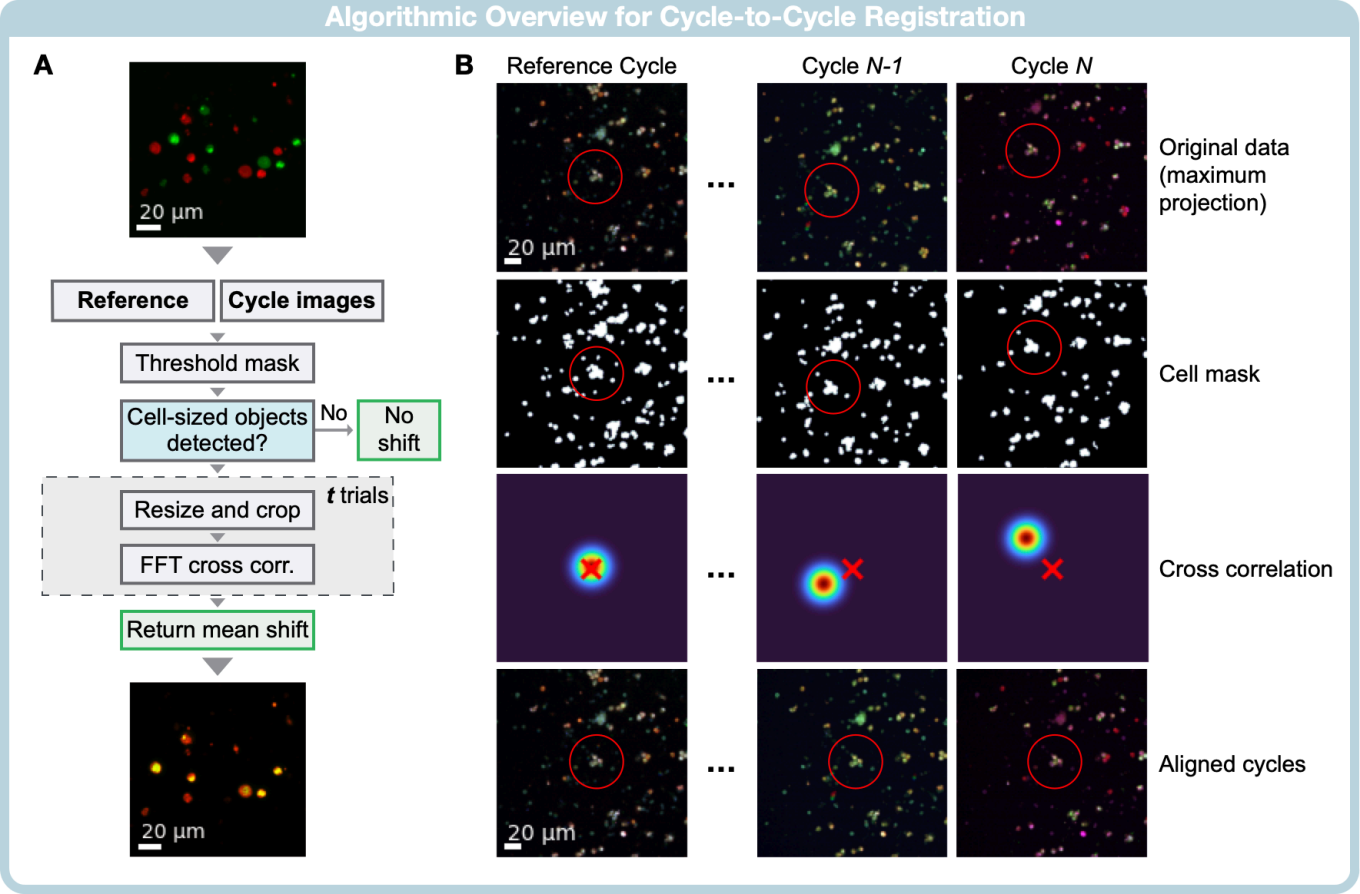
Correction of inter-cycle image offsets. **(A)** Image registration algorithm. A single specimen undergoes *N* imaging cycles, each cycle producing *m* FOVs. One cycle is chosen as the “reference” to which all other cycles are aligned. For a single FOV, cycles are collapsed by maximum projection, and a threshold mask is computed to roughly determine the presence of cell-sized objects. Each image is then downsampled and randomly cropped. Finally, the masked cross-correlation is computed over *t* trials, and the mean shift is returned. **(B)** Visual examples for cycle registration. The top row shows the original data after maximum projection. The second row demonstrated the threshold mask used to search for cell-sized objects. The heat maps in the third-row show where the correlation between the current cycle and the reference image is highest. The final row shows the aligned images. Red circles highlight a cell cluster of interest. In the final row, this cluster is in the same position for all cycles.

Figure 2A illustrates the CycloNET image registration algorithm. The algorithm processes image datasets derived from several staining cycles (denoted as *N* cycles). Multiple fields of view (FOVs) are collected for each staining cycle to capture as many cells as possible. In this scenario, each FOV tile set can be represented as FOV_*i*,*j*_, where *i* indicates an individual field of view (*i* = 1, 2, …, *M*) and *j* indicates the particular cycle number (*j* = 1, 2, ..., *N*). We found that images from any two cycles had similar spatial offsets across all FOVs, allowing us to align full cycles by using a single FOV per cycle. Thus, the FOV numbers to be processed for alignment is reduced from (*N* × *M* × *C*) to just (*N* × *C*), where *C* is the number of fluorescent channels used in microscopy. To further minimize dataset size and improve accuracy in low-signal images, the *C*-channel fluorescent signals were normalized and collapsed by maximum projection, resulting in a set of *N* images. By using the maximum projection across individual channels, we enhanced the visibility of cells, as some cells might be visible in one channel and not another, providing more “anchoring points” for accurate alignment. Finally, a reference is selected among these *N* collapsed images, to which all other cycles are aligned. These are labeled in Fig 2A as “Reference” and “Cycle images.”

The translations between the N images were calculated by masked cross-correlation. This strategy reduced the impact of cellular debris by focusing only on cell-shaped foreground objects specified by the cell mask (see **Methods** for details). At the time of cross-correlation calculation, images were also downsampled two-fold and randomly cropped to further reduce dataset size. This process was repeated for some ***t*** trials, with different random crops at each trial. Finally, the mean shift (after removing outliers) is calculated and assigned to all FOVs in a given staining cycle.

Figure 2B shows snapshots of the image registration process. The maximum projection across individual channels increased the number of cells available as anchoring points for the cross-correlation algorithm. One such anchoring point is indicated by red circles, demonstrating how a cluster of cells shifts to overlap with the reference cycle after alignment. We tested our algorithm on a set of five human specimens (FOVs per sample: 20, 24, 35, 30, 36). On average, a single field of view with seven imaging cycles was aligned in 42 seconds, while a full specimen containing ~20 FOVs typically aligns in under 6 minutes using an Intel^®^ Core^™^ i7–6850K processor. Alignment failed in 5% of 142 FOVs, typically in FOVs with very low contrast in the reference signal or low cellularity.

### Cell segmentation and validation

**Figure 3.**
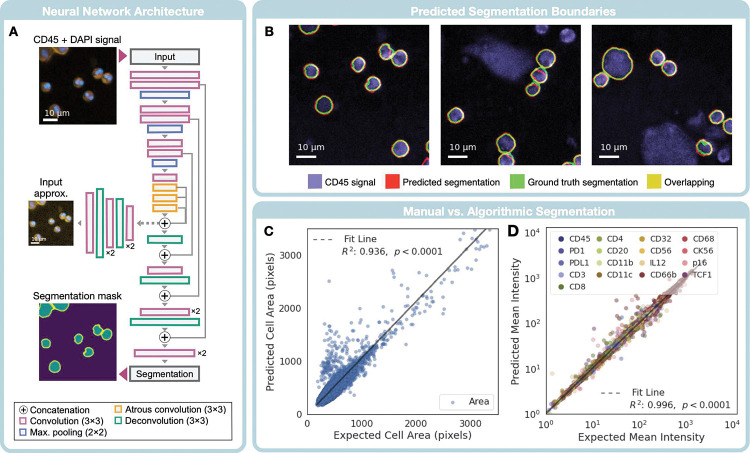
Single-cell segmentation by a neural network. **(A)** Neural network architecture. The CD45 and DAPI (nuclear) stains were used as the input to a modified U-NET architecture to produce a 3-class segmentation mask. The bottleneck features were also used for a secondary auto-encoding task. **(B)** Segmentation boundaries. Green and red boundaries, respectively, represent cell boundary pixels by manual and CycloNET segmentations. Yellow represents the overlap between the two methods. **(C)** Functional comparisons. (Left) A comparison is made between the areas of manually segmented cells and neural network-predicted areas. (Right) Similarly, the mean fluorescent signal per cell was compared under manual and algorithmic segmentation.

Following alignment, CycloNET generated segmentation masks for individual cells by applying a pre-trained neural network. To detect immune cells in the sample, the algorithm was trained on image stacks consisting of CD45 and nucleus (DAPI) channels, as described in Fig. 3A. Using a modified U-Net structure, we designed a neural network to produce a 2-dimensional array where each pixel was labeled as either: i) background, ii) cell interior, or iii) cell boundary^20^ (Fig. 3A). The encoded features used to construct segmentation masks were regularized by secondary auto-encoding and cell-counting tasks^21^, resulting in a loss function,

$$L=L_{seg}+\lambda_{c}\cdot L_{count}+\lambda_{a}\cdot L_{auto}+\beta \cdot L_{reg}=\left(L_{ce}+\alpha \cdot L_{jacc}\right)+\lambda_{c}\cdot L_{count}+\lambda_{a}\cdot L_{auto}+\beta \cdot L_{reg}$$

where the segmentation loss, $L_{seg}$, was split into the cross entropy loss ($L_{ce}$) for pixel-by-pixel classification and the per-class Jaccard loss metric $L_{jacc}.\;L_{count}$ and $L_{auto}$ represent the mean squared error loss metrics for the cell counting and autoencoding tasks, respectively. Finally, $L_{reg}$ represents the L2 regularization loss applied to all weights in the network, and $\alpha ,\;\lambda_{c},\;\lambda_{a},\;\beta$ assign weights to each loss type. The network was trained using the CD45 and nuclear stains for 17 human specimen FOVs (See Supp 2). At the end of the training, the average F1 score among all pixel classes was 0.94. A single FOV was scanned for CD45+ immune cells in 17.9 seconds (*n* = 134).

Figure 3B shows the comparison between manual and CycloNET segmentations. Defining the cell boundary class was found to be crucial for separating cells in contact with each other. To evaluate the functional performance of our segmentation network, we interrogated the correlation between biomarker values after automated and manual segmentation on the test set. Among all 134 image FOVs in our test group, the correlation for the number of cells counted was 0.92, with a 0.11 false positive and 0.13 false negative cell detection rate. In single cells, the correlation in cell area was 0.936 (Fig. 3C), and the correlation for mean fluorescent intensity was greater than 0.996 for all markers (Fig. 3D).

### Single-cell profiling and clustering in patient samples

**Figure 4.**
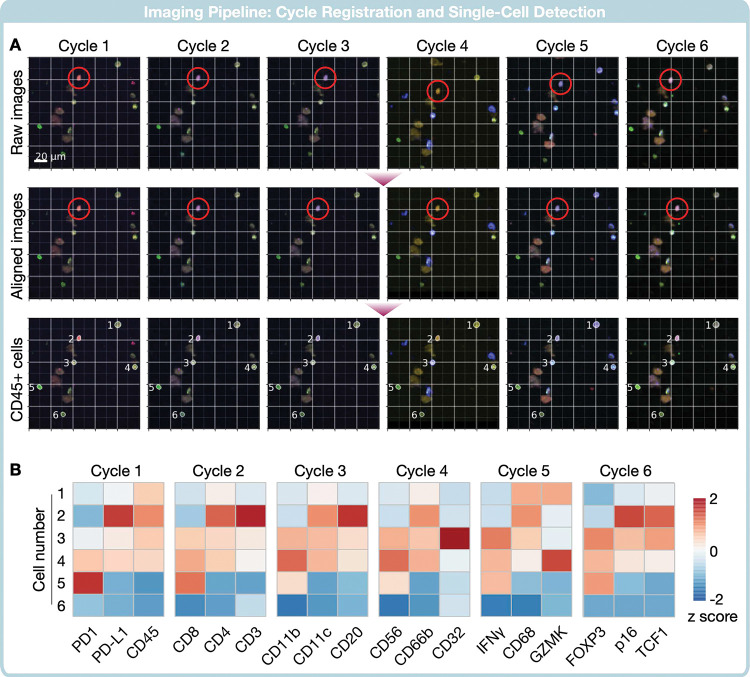
Fluorescence Image stack to single-cell metrics. **(A)** We visualize the full image stack of fluorescent signals as single-cell data is extracted. (Top row) For each cycle, we visualize 3 biomarker signals in an RGB image. A single immune cell is highlighted by a red circle, which is at different positions in each imaging cycle. (Middle row) The image stacks are aligned for inter-cycle translations. (Bottom row) Among the many cells seen in the window, immune cells are segmented by the neural network. Each cell is highlighted by a white border and a numeric ID. **(B)** The fluorescent signal within each cell’s interior is mean aggregated, and a z score is calculated for all cells. The heat map shows each cell’s relative signal strength for each of the 18 markers.

We finally applied the entire CycloNET process to an example patient dataset. The input data were taken from seven cycles of staining (Fig. 4A, top row), with each cycle probing three protein markers found in immune cells. The algorithm corrected spatial shifts between cycles (Fig. 4A, middle) and segmented immune cells based on CD45 and nucleus stains (Fig. 4A, bottom). Single-cell masks were then applied to the aligned image stacks to extract marker expression in individual cells (Fig. 4B).

**Figure 5.**
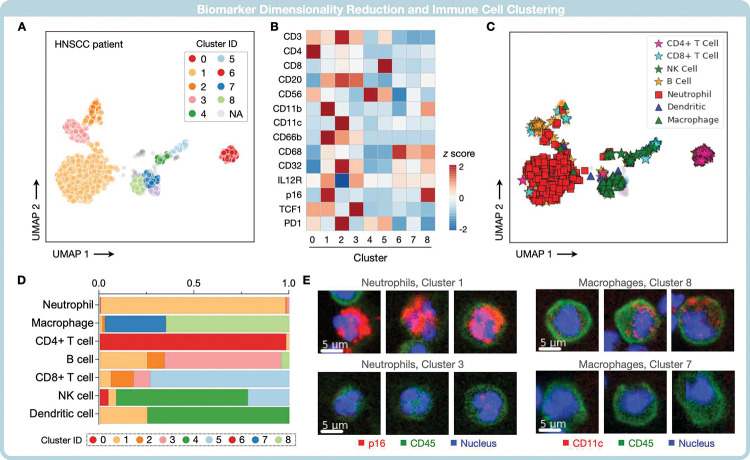
Patient data visualization. **(A)** Dimensionality reduction. The multi-dimensional single-cell dataset generated from a patient sample was projected onto a two-dimensional manifold by the UMAP algorithm. Cells were clustered by the unsupervised HBDSCAN algorithm. **(B)** Mean marker intensities were calculated for each cluster, and relative marker intensities were visualized in a heat map. **(C)** Common immune cell types were overlaid to individual cells in existing clusters. **(E)** CycloNET subclassified single-cell types according to their distinct phenotypes. For a given immune cell type, its relative presence among unsupervised clusters is summarized. **(D)** Example images show macrophages and neutrophils belonging to different unsupervised clusters.

To further aid in interpreting the large single-cell dataset, we incorporated dimensionality reduction and an unsupervised clustering protocol. Dimensionality reduction was performed by the uniform manifold approximation and projection (UMAP) algorithm, and potential subpopulation clusters were identified by the Hierarchical density-based spatial clustering of applications with noise (HDBSCAN) algorithm (see Methods for details)^22, 23^. Figure 5 shows an exemplary profiling result of an HNSCC FNA specimen. CycloNET identified 1528 immune cells in this sample and generated single-cell data for 17 protein markers. After dimensionality reduction, a total of 9 distinct clusters were assigned by the HDBSCAN algorithm (Fig. 5A). Each cluster was found to have a unique biomarker profile based on average intensities (Fig. 5B).

We investigated the detected clusters by identifying common immune cell phenotypes (Table 1). Neutrophils and macrophages were among the most abundant, while natural killer and dendritic cells were the least present (Fig. 5C). Some of the manually defined phenotype assignments (e.g., CD4+ T cells) closely matched existing clusters, while others spread across different clusters (e.g., macrophages). A close look revealed different phenotypes in a nominal cell type (Fig. 5D). For example, neutrophils were found in clusters 1 and 3, separated mainly by p16 expression. Similarly, a sizable percentage of macrophage cells were found to differ in CD11c expression (Fig. 5E). Overall, the results demonstrate CycloNET’s capability of streamlining multi-dimensional single-cell phenotyping.

## CONCLUSIONS

High-throughput, multiplexed microscopy generates expansive data (>giga bytes) that are often beyond manual inspection. The developed CycloNET can simplify large-scale dataset analysis and extract biological information. CycloNET integrated three key modules: i) a custom image-alignment algorithm designed to minimize errors and computation time; ii) a trained neural network to provide consistent, accurate segmentation masks for target cells while avoiding cellular debris or non-target cell types; and iii) a dimensionality-reduction to visualize the multi-dimensional single-cell data. Equipped with these technical capacities, CycloNET efficiently processed gigapixels of imaging data (>5,000 cells) and produced a single-cell dataset, all in under 2 minutes. The data produced by CycloNET further revealed sub-clusters within conventional immune cell phenotypes.

The neural network that identifies single cells is easily adapted to different tasks. In the presence of novel datasets, the pre-trained network can be re-trained from scratch, fine-tuned, or even combined with other networks. In future studies, we will explore techniques to facilitate transfer learning so that researchers may capture the full mosaic of different sample structures and cell types. We can also explore methods to capture sub-cellular information more efficiently. For example, per single cell, high-resolution details such as the location and concentration of fluorescent staining are lost when utilizing averaging metrics. By contrast, an intelligent state-of-the-art auto-encoding scheme could summarize morphologic information with little supervision. With this expansion in CycloNET’s analytical power, researchers will be better equipped to understand underlying biological mechanisms, improving diagnosis and prognosis metrics and potentially leading to new therapeutic approaches.

## METHODS AND MATERIALS

### Human tumor biopsy dataset and image acquisition

FNA samples from HNSCC patients were collected, stained, and imaged as previously reported^19^. The study protocol was reviewed and approved by the Institutional Review Board of Massachusetts General Hospital (IRB number 2014P000559), and the overall procedures followed institutional guidelines. Informed consent was obtained from newly diagnosed and recurrent/metastatic head and neck squamous cell carcinoma (HNSCC) patients, and samples were acquired between Jan 1, 2020 and June 30, 2021. Briefly, samples were collected by aspiration, fixed, and serially stained with three protein markers per cycle, followed by a signal quenching step. This process was repeated until all marker signals (and corresponding quenched background signals) were collected. For each specimen, 20–40 fields of view were imaged by an Olympus BX-63 upright automated epifluorescence microscope. To develop the three modules in this computational pipeline, a total of 17 human samples were collected for training and validation and 5 samples for final evaluation. For analysis, collected images were processed using a graphic user interface (GUI) developed with the Qt GUI toolkit for Python^24^.

### Image pre-processing

Acquired raw images were preprocessed for background subtraction, and imaging cycles were aligned together. To detect immune cells, the CD45 signal, along with a corresponding DAPI image, was processed by a pre-trained neural network to generate single-cell outlines. Once biomarker intensities were recorded for each cell, cell-type labeling for major immune groups was conducted. For each specimen, quenched background signals were subtracted from corresponding fluorescence signals pixel-by-pixel. Pixel intensity for each signal was then normalized using the 25th and the 99th percentiles as lower and upper limits. Next, a Gaussian filter was applied to reduce background impulse noise.

### Image alignment

An image registration step was applied between all cycles in a single field of view for a given specimen to account for small translations between imaging steps. First, a reference cycle was chosen for each sample with the provided user interface (Fig. 2A). The general registration algorithm structure is outlined in Figure 2B. Signals from the same imaging cycle were collapsed by maximum projection and rescaled by a factor of 0.5. Next, a foreground mask was produced to roughly detect cell-sized objects. Fields of view with no few cell-sized objects were removed from the analysis. Finally, the collapsed signals and corresponding masks were randomly cropped by a factor of 0.75, and the translation shift was calculated by masked cross-correlation using the Python Sci-kit Learn library. The resulting translation was then stored and averaged over three trials to minimize translation errors resulting from signal noise. Finally, a single-cycle translation was applied to all fields of view.

### Cell segmentation

A set of 17 human specimen fields of view were used for training and 6 for validation. Each field of view was tiled into 300 × 300 arrays with 20% overlap for each sample. To reduce class imbalance, examples with no labeled cells were removed from the training dataset. The final dataset sizes for training and testing were 7450 and 6302, respectively. The hyper parparameters, *α*, *λ*_*c*_, *λ*_*a*_, *β,* were set to 1 × 10^3^, 1 × 10^3^, 5 × 10^−1^, and 1 × 10^−4^, respectively. The neural network was defined using the Tensorflow library, trained on two NVIDIA GTX 1080 Ti GPUs for 700 epochs with a batch size of 16, using the Adam optimizer with a learning rate of 10^−5^. Additionally, stochastic i) 90° rotation, ii) horizontal and vertical flipping, iii) gaussian noise, and iv) mean shift for data augmentation was employed in the training steps. The best-performing model was saved over five trials, as determined by pixel classification accuracy. To adjust for background-class imbalance, we weighted the cross entropy loss for cell interior (class ii) and cell boundary (class iii) by 3-fold importance compared to the background. Misclassifications of boundary pixels as cell interior were further weighted with two-fold importance to encourage the network to prioritize cell boundaries and avoid merging neighboring cells.

### Clustering

CD45-positive immune cell segmentation masks were applied to aligned fluorescent signals to aggregate fluorescent intensity for each marker on a single-cell basis. The aggregate signal was then averaged over each segmented cell area. High-intensity outliers were removed by mean absolute deviation, aggregate intensities were log-transformed, and positivity thresholds for each marker were set to 3 standard deviations below the signal mean. Different immune cell populations were defined based on the expression level of a combination of markers, as in Table 1. Dimensionality reduction was then applied to the sample’s single-cell data by applying uniform manifold approximation and projection (UMAP) using the Sci-kit Learn library for Python and optimized to minimize the distance between predefined cell phenotypes^23, 25^. Finally, to define distinct cell clusters, we applied the hierarchical DBSCAN algorithm, a density-based approach that can detect clusters of arbitrary shape^22^.

## Figures and Tables

**Table 1.** Marker combinations for immune cell phenotyping

Cell type	Markers	
	Positive	Negative

Immune cells	CD45	CK56
CD4+ T cells	CD45, CD3, CD4	CD11b
CD8+ T cells	CD45, CD3. CD8	CD11b
Natural killer cells	CD45, CD56	CD3, CD11b
B cells	CD45, CD20	CD3, CD11b
Neutrophils	CD45, CD11b, CD66b	CD3
Macrophages	CD45, CD11b, CD68	CD3
Dendritic cells	CD45, CD11b, CD11c	CD3

## Data Availability

Imaging data that support the findings of this study are available from the corresponding authors on reasonable request, subject to approval from the Institutional Review Board of the Massachusetts General Hospital. Source codes for the image analysis will be available (https://csb.mgh.harvard.edu/bme_software) upon the publication of the current manuscript.
